# Effect of Chinese herbal medicine for patients with benign thyroid nodules in adults: A protocol for systematic review and meta-analysis

**DOI:** 10.1097/MD.0000000000024591

**Published:** 2021-02-26

**Authors:** Shuang Ouyang, Weihong Li, Peng Yu, Han Li, Haiyang Cai, Jing Wu

**Affiliations:** Basic Medical College, Chengdu University of Traditional Chinese Medicine, Chengdu, China.

**Keywords:** Chinese herbal medicine, protocol, systematic review, thyroid nodules

## Abstract

**Background:**

Thyroid nodules (TN) are discrete lesions within the thyroid gland and are a common clinical problem detected in 19% to 68% of people. TN are more common as age increases and occur more frequently in women. TN can cause pressure symptoms, cosmetic complaints, and thyroid dysfunction. Treatment for benign thyroid nodules includes thyroid hormone therapy, surgery, radioiodine treatment, percutaneous ethanol injection therapy, and laser or radiofrequency treatment to shrink nodules. In China and many other countries, doctors use Chinese herbal medicines (CHM) to treat TN. However, systematic review and meta-analysis has not been found to assess the effects and safety of CHM in curing TN at present. Hence, the systematic review is conducted to scientifically and methodically evaluate the value of its effectiveness and safety of CHM on TN.

**Methods:**

Literatures related to CHM for TN from the establishment of the database to November 2020 will be retrieved from the following databases: PubMed, Excerpta Medica Database (EMBASE), MEDLINE, Web of Science, Cochrane Library, SpringerLink, WHO International Clinical Trials Registry Platform (ICTRP), Wanfang Database, China National Knowledge Infrastructure (CNKI), Chinese Biomedical Literature Database (CBM), and Chinese Scientific Journal Database (VIP). There are no language restrictions for retrieving literatures. Case reports, animal studies, editorials, expert opinions, reviews without original data, and studies on pediatric population were excluded. Eligible randomized clinical trials (RCTs) evaluating the effectiveness and safety of CHM in TN patients will be put in the study including nodule volume reduction ≥50%, pressure symptoms, cosmetic complaints, quality of life, and adverse events. By scanning the titles, abstracts and full texts, 2 reviewers will independently select studies, extract data, and assess the quality of study. Meta-analysis of RCTs will be conducted using Review Manager 5.1 software. The results will be presented as risk ratio for dichotomous data, and standardized or weighted mean difference for continuous data.

**Result:**

This study will provide high-quality available evidence for the treatment of TN with CHM based on nodule volume reduction ≥50%, pressure symptoms, cosmetic complaints, quality of life, and adverse events.

**Conclusion:**

The systematic review will to evaluate the efficacy of CHM in treating benign thyroid nodules in adults and provide evidence for clinicians.

**INPLASY registration number:**

INPLASY2020120093.

## 1 Introduction

A thyroid nodule is a discrete lesion within the thyroid gland that is palpable and ultrasonographically distinct from the surrounding thyroid parenchyma.^[1]^ TN are divided into cysts, inflammatory nodules, and tumoural nodules (benign, malignant) and may present as proliferative nodular goiter.^[2]^ TN, whether solitary or multiple, are a common clinical problem. Epidemiologic studies have shown the prevalence of palpable TN to be approximately 5% in women and 1% in men living in iodine-sufficient parts of the world.^[3,4]^ In contrast, high-resolution ultrasound (US) can detect TN in 19% to 68% of randomly selected individuals, with higher frequencies in women and the elderly.^[5,6]^ The incidence of TN varies among different populations around the world. In iodine-sufficient areas, for instance, palpable TN are found in about 4% to 7% of the population, and they are even more prevalent among individuals living in areas of iodine deficiency.^[7]^ TN are more common as age increases and as iodine intake decreases, and they occur more frequently in women.^[8]^ In all, 3% to 7% of the population with palpable thyroid nodules has been found in China; however, high-resolution ultrasonography can detect thyroid nodules in 20% to 70% of the population.^[2]^ As a result, we are now facing an “epidemic” of TN. Most TN are asymptomatic, and people often find them incidentally on physical examination or self-palpation or on imaging studies performed for unrelated reasons. A minority of patients with TN have thyroid dysfunction, and some patients with TN show obstructive symptoms.^[9]^ The clinical importance of TN rests with the need to exclude thyroid cancer, which occurs in 7% to 15% of cases depending on age, sex, radiation exposure history, family history, and other factors.^[7,10]^ Thyroid malignancy may be associated with the following clinical features:

1. Historical features: young (younger than 20 years of age) or old (older than 60 years of age) age, male sex, neck irradiation during childhood or adolescence, rapid growth, recent changes in speaking, breathing or swallowing or family history of thyroid malignancy or multiple endocrine neoplasia type 2;
2. Physical examination findings: firm and irregular consistency of nodules, fixation to underlying or overlying tissues, vocal cord paralysis or regional lymphadenopathy;
3. Ultrasound findings: hypoechoic lesions, irregular margins, presence of calcifications, absence of halo or internal, or central blood flow; and
4. Low suspicion: echofree (cystic) lesions or homogeneously hyperechoic lesions.^[11]^

Thyroid-stimulating hormone (TSH) measurements, thyroid ultrasound and fine-needle aspiration biopsy (FNAB) are the key examinations to distinguish benign and malignant lesions. The diagnosis of thyroid nodule malignancy is established through history and physical examination, followed by ultrasonography, FNAB and evaluation of the sample by an experienced cytologist.^[7]^ With the discovery of a thyroid nodule larger than 1 to 1.5 cm in any diameter, one may use serum TSH and free thyroid hormone concentrations as a first-line screening test.^[11]^ With an elevated TSH level, measurement of serum antithyroid peroxidase (anti-TPO) antibody and antithyroglobulin (anti-Tg) antibody levels may be helpful for diagnosis of chronic autoimmune thyroiditis.^[5]^ If the serum TSH is lower than normal, a radionuclide thyroid scan should be performed to prove whether the nodule is functioning, shows dysfunction (“warm”) or is non-functioning (“cold”). Functioning nodules rarely harbour malignancy.^[12]^ Calcitonin may be a useful serum marker of medullary thyroid carcinoma.^[13]^ A baseline serum calcitonin value of 10 to 100 pg/ml is abnormal (normal baseline <10 pg/ml) and should result in further investigation; values that exceed 100 pg/ml are highly suggestive of medullary thyroid carcinoma.^[14]^ In the initial diagnosis of thyroid malignancy, computerized tomography (CT) scanning and magnetic resonance imaging (MRI) can not provide higher quality images of the thyroid and cervical nodes than ultrasonography (US). US examination is highly accurate and sensitive in evaluating thyroid nodules.^[15–17]^ CT examination of the lower central neck is preferable when tracheal or mediastinal invasion is suspected.^[7]^ FNAB of thyroid nodules has eclipsed all other techniques for diagnosing thyroid cancer, with reported overall rates of sensitivity and specificity exceeding 90% in iodine-sufficient geographical areas.^[11]^ Despite many investigation options available to the clinicians, the management of TN essentially depends on the FNA results.^[15]^ The possible reports are benign, malignant, suspicious malignancy, atypia of undetermined significance or follicular lesion of undetermined significance, follicular neoplasm or suspicious for a follicular neoplasm, and non-diagnostic.^[18]^ Beside cytological diagnosis, the treatment plan may depend on the patient's age and gender and the characteristics of the nodule (size, consistency, activity, and number), and whether it is functioning or not.^[15,19–21]^ Asymptomatic benign euthyroid nodules need only watchful follow-up with annual or biannual US and TSH testing.^[15]^ Nevertheless, on some occasion, benign thyroid nodules do require therapeutic intervention, especially when they grow large and when they cause obstructive symptoms.^[21–24]^ Any type of malignancy, or when malignancy cannot be ruled out, indicated the need for surgical resection. The scope of surgery depends on the type of tumor. Simple hemithyroidectomy may be recommended as the initial step for follicular neoplasm or as the definitive treatment for a small, isolated papillary carcinoma if no other risk factors are present.^[25]^ All other thyroid malignancy deserve total thyroidectomy with complementary local node resection. If the histology of the follicular neoplasm confirms the presence of carcinoma, total thyroidectomy should be completed.^[15,23,25]^

In China and many other countries, doctors use Chinese herbal medicines (CHM) to treat many diseases, including TN. Traditional Chinese medicine (TCM) in the prevention and treatment of TN has various methods, rich theoretical experience, significant advantages, little adverse reactions, and a long history. It can avoid adverse reactions such as hypothyroidism, local tissue damage, and severe pain caused by surgery and other traumatic treatment, and help to alleviate the psychological pressure of patients.^[27]^ The contents of traditional Chinese herbal preparations are variable depending on TCM syndromes of patients. CHM for the treatment of TN include Milkvetch, Codonopsis Pilosula, Figwort root, Pangolin Scales, Selfheal, Chinese Thorowax root, Nutgrass Galingale Rhizome, Seaweed, Laminaria Tents, TCM holds that the occurrence of TN is related to environment, diet, and emotion. The treatment of benign thyroid nodules is based on its etiology, pathogenesis, and treatment rules. Most of the prescriptions are combined with self-made prescriptions, and drugs such as resolving phlegm and softening, soothing the liver and regulating qi, activating blood and removing blood stasis, clearing heat, and tonic drugs are often used. Clinical studies from the Chinese literature show that Chinese herbal preparations might shrink TN without producing significant adverse effects.^[26]^ According to the theory of Chinese medicine, practitioners recognize that TN are caused by blood stasis, Qi stagnation and phlegm coagulation. Several explanations have been offered for the effects of CHM in inhibiting the proliferation of TN cells:

1. decreased sensitivity of thyroid nodule cells to TSH;
2. decreased activity of TSH;
3. induced apoptosis of TN cells; and
4. direct injury of TN cells.^[26]^

Herbal preparations are prescribed by practitioners on the basis of patients’ symptoms and observations of the tongue and pulse, and this accounts for the great variation seen in the use of herbal preparations.

Treatments are made into a Chinese proprietary medicine or a compound of several herbs, irrespective of preparation. Besides the traditional herbal decoction (remaining liquid prepared by boiling a mixture of different herbal medicines), various forms of herbal medicines may be used, such as patent medicines (fixed formula of Chinese medicines in different forms, such as granules, particles, tablets, capsules, or liquids)^[27]^ and extracts of herbal medicine,^[28]^ for example, Selfheal oral liquid (liquid prepared by boiling Selfheal).^[9]^ Up until now, many published studies have described the effects of CHM in the treatment of thyroid nodules.^[26]^ In 2017, Chen systematic review provided evidence for the treatment of TN with CHM, proving the effects of CHM in the treatment of TN.^[29]^ In 2017, Li systematic review provided the regularity of CHM in the treatment of TN on the basis of analyzing the frequency of effective compound prescription of CHM in treating thyroid nodules in recent years.^[30]^ The clinical research of “Xiao ying san jie” fang on treating TN provided the effect of CHM in treating TN.^[31]^ Xie observed the clinical efficacy of Xiao-Ying-Ruan-Jian-Tang in the treatment of TN and explored its mechanism of action from both theoretical and clinical aspects.^[32]^

However, the efficacy and safety of CHM in treating TN are still unclear due to the large number of TCM prescriptions. No systematic review has yet examined CHM for the treatment of benign thyroid nodules in adults. The purpose of this study is to systematically review the available literatures on the therapeutic effect of CHM on TN and provide evidence for clinicians.

## 2 Methods

### 2.1 Registration

This study has been registered in the International Platform of Registered Systematic Review and Meta-analysis Protocols (INPLASY.COM) with the registration number of INPLASY2020120093. This protocol adheres to the preferred reporting items for systematic reviews and meta-analyses protocols 2015.^[33]^

### 2.2 Types of study

We will put all RCTs related to CHM for TN into the study of the effectiveness of CHM for TN.

### 2.3 Participants

Participants must be imaging-confirmed TN, and aged 18 years and older. Participants with malignant thyroid nodules, infection, and other pathologic change was not taken in consideration in this study.

### 2.4 Interventions

Experimental interventions: Chinese herbal medicines (CHM).

Control interventions: placebo, blank control, and conventional medicine (such as levothyroxine).

CHM included all type of herbal medicine. There is no restriction of dosage, frequency, administration method, or duration of treatment. The usage of western medicine in the control group should be consistent with that in the experimental group.

### 2.5 Outcome

The primary outcomes include:

1. Nodule volume reduction ≥50% (evaluated by ultrasonography measurements);
2. Pressure symptoms, cosmetic complaints or both;
3. Adverse events.

The secondary outcomes include:

1. Quality of life (measured by a validated instrument);
2. Cancer occurrence;
3. Changes in number and size of TN.

### 2.6 Data sources

#### 2.6.1 Electronic searches

We will screen the comprehensive literature from relevant electronic databases, including 7 English databases, consisting of PubMed, Excerpta Medica Database (EMBASE), MEDLINE, Web of Science, Cochrane Library, SpringerLink, and WHO International Clinical Trials Registry Platform (ICTRP), and 4 Chinese databases, namely Wanfang Database, China National Knowledge Infrastructure (CNKI), Chinese Biomedical Literature Database (CBM), and Chinese Scientific Journal Database (VIP). All the RCTs will be collected from the database establishment to November 2020. The search items include benign thyroid nodules, thyroid benign nodules, thyroid nodules, herbal, drug therapy, herbal treatment, traditional treatment, used alone or in combination to ensure the same searching terms in both Chinese and English database, an equivalent translation of the search terms will be adopted. The detailed selection process for PubMed is presented in Table 1.

**Table 1:** Search strategy used in PubMed database. This search strategy will be modified as required for other electronic databases.

Number	Search terms
1	exp Chinese medicine
2	traditional Chinese medicine. ti, ab.
3	proprietary Chinese medicine. ti, ab.
4	Chinese herbal medicine. ti, ab.
5	or 1–4
6	exp thyroid nodules
7	thyroid nodules. ti, ab.
8	benign thyroid nodules. ti, ab.
9	or 6–8
10	randomized controlled trial. pt.
11	controlled clinical trial.pt
12	randomized. ab.
13	randomly. ab.
14	trial. ab.
15	or 10–14
16	exp animals/not humans.sh.
17	15 not 16
18	5 and 9 and 17

#### 2.6.2 Searching other resources

Additionally, relevant meeting minutes and qualified research references also will be searched manually.

### 2.7 Data selection

**Figure 1:**
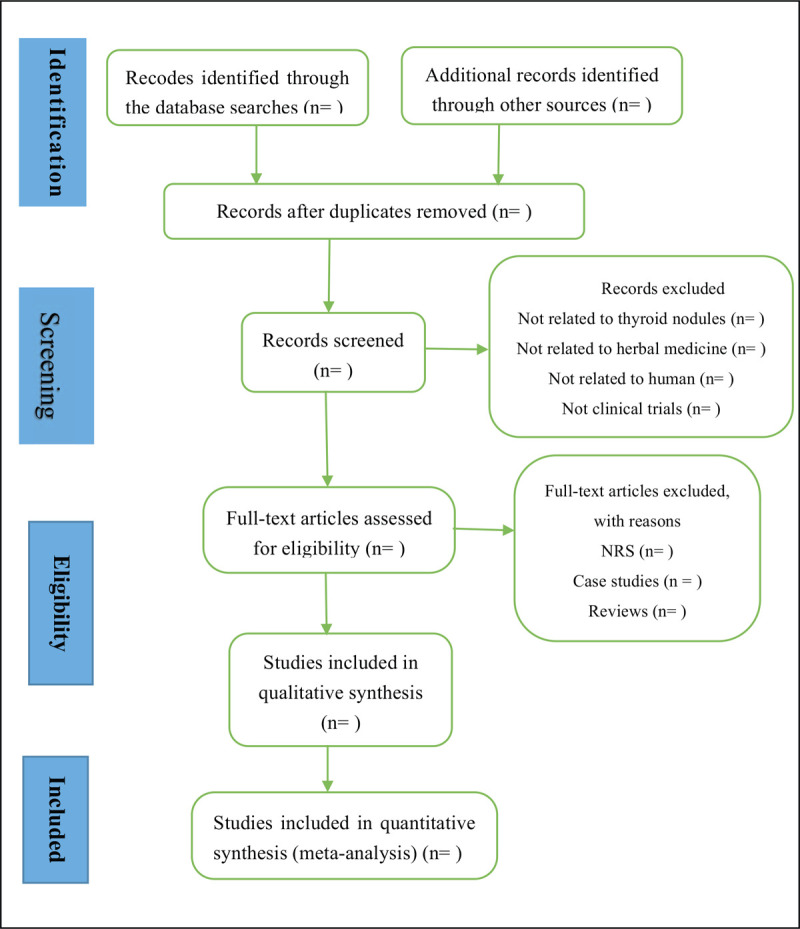
Flow diagram of studies selection process.

We will organize a session for all reviewers to learn relative information of the study for insight on the purpose and process of the study, and the relevant teacher has been trained and gained certifications in Chinese Cochrane Centre. Two reviewers independently investigate all relevant literatures and screen from title to abstracts respectively to extract eligible articles, then exclude repeated. Subsequently, reviewing the full-text and comprehensively considering to identify eligible studies. The all studies that reviewers chosen will be discussed in the group until the final team consensus reached. Disagreements between the reviewers will be settled after group discussion with a third reviewer or expert. If the information is insufficient or unclear, we will contact the corresponding author to ask for more information or clarification via email or phone. Data extraction items included: the first and corresponding author, year of publication, clinical diagnostic information, course of disease, sample size, age of participants, intervention details, control and outcome, treatment time, duration of follow-up, and adverse events. Disagreements will be settled after group discussion and if necessary, consulting experts and arbiter. The flow chart is tabulated in Figure 1.

### 2.8 Quality assessment

The assessment of potential bias risk and quality will be executed with 2 reviewers by the Cochrane Handbook 5.1.0 bias risk assessment tool, including random sequence generation, allocation concealment, the blinding 000method of participants, researchers, outcome assessment, incomplete result data, selective outcome reporting, and other issues. This the bias risk will take L (low risk), U (unclear risk), and H (high risk) to represent the assessment results.

### 2.9 Data synthesis

The differences between the intervention group and the control group will be evaluated. The continuous data, we will adopt the mean difference (MD) with 95% confidence intervals (CIs) to analysis the therapeutic efficacy. The other forms of data will be converted into MDS. The standard MD of 95% CIs will be used for the outcome variables of different scales. The other binary data will be converted into relative risk (RR) values. With respect to binary data, we will adopt the RRs with 95% CIs to analysis the processing effect. The meta-analysis studied in this review will be performed using the Cochrane Collaboration's software program Review Manager v.5.3.5 for Windows. For research with insufficient or missing data, the 2 reviewers (Han Li and Jing Wu) will contact corresponding authors via email or phone to integrate and verify data as far as possible.

### 2.10 Assessment of heterogeneity

Heterogeneity of the including studies will be analyzed by Chi-Squared test and evaluated quantitatively by *I*^2^ test. If heterogeneity is not statistically significant (*P* > .1 and *I*^2^ < 50%), a fixed-effect model will be adopted for meta-analysis. If heterogeneity is statistically significant (*P* < .1, *I*^2^ > 50%), a random-effect model can be employed.

### 2.11 Subgroup analysis

If there are a plenty of subgroup studies, subgroup analysis will be conducted to detect the heterogeneity among groups. We will take treatment time or dose of CHM, different forms of herbal medicine, duration or severity of TN, syndrome differentiation, formulations, sex and age of patients into account.

### 2.12 Sensitivity analysis

Sensitivity analysis will be conducted to identify the robustness of the conclusions. The main decision nodes include methodologically quality, sample size, missing data. Studies that are not randomly generated to improve conclusions will be excluded.

### 2.13 Reporting bias

If a result of a meta-analysis contains >10 articles and above, the risk of publication bias will be evaluated by a funnel plot.

### 2.14 Ethics and dissemination

The systematic has no necessary to acquire the ethical approval, because we are not involved in the data related to individual or private message. In addition, the findings will be shared for peer-reviewed journals publications and presentations at conferences or relevant meeting for the clinician.

## 3 Discussion

Epidemiologic studies have shown the prevalence of palpable thyroid nodules to be approximately 5% in women and 1% in men living in iodine-sufficient parts of the world.^[3,4]^ In contrast, high-resolution ultrasound (US) can detect thyroid nodules in 19% to 68% of randomly selected individuals, with higher frequencies in women and the elderly.^[5,6]^ Therefore, we are now facing an “epidemic” of thyroid nodules.^[9]^ Established treatment for thyroid nodules, such as levothyroxine, radioiodine, ethanol injection, and surgery, there are many adverse reactions and the long-term effect is not good. It is a fact that more and more patients with TN are seeking alternative treatment. CHM has been used to treat TN for more than 2000 years. Clinical studies from the Chinese literature show that Chinese herbal preparations might shrink thyroid nodules without producing significant adverse effects.^[26]^ Therefore, it is necessary that systematic reviews and meta-analyses be used to assess the effect and safety of Chinese herbal medicine in the treatment of benign thyroid nodules. The results of this study will be a comprehensive review of CHM treatment and provide basis for clinical gynecologists to use Chinese herbal medicine to treat benign thyroid nodules.

## Author contributions

**Conceptualization:** Shuang Ouyang, Weihong Li.

**Formal analysis:** Shuang Ouyang.

**Investigation:** Peng Yu, Han Li.

**Methodology:** Haiyang Cai, Jing Wu.

**Project administration:** Shuang Ouyang, Weihong Li.

**Supervision:** Jing Wu, Haiyang Cai.

**Validation:** Weihong Li.

**Writing – original draft:** Shuang Ouyang.

**Writing – review & editing:** Shuang Ouyang.
